# A prospective study for validating an automated AI-based system for detecting age-related macular degeneration in clinical settings

**DOI:** 10.1038/s41598-025-94866-6

**Published:** 2026-07-29

**Authors:** Alauddin Bhuiyan, Arun Govindaiah, Oscar Otero-Marquez, Anna Fabczak-kubicka, Tasin Bhuiyan, Katy Tai, Avnish Deobhakta, Theodore Smith

**Affiliations:** 1 iHealthscreen Inc, New York, USA; 2 New York Eye and Ear Infirmary at Mount Sinai, New York , USA; 3 New York University, New York, USA

**Keywords:** Age-related macular degeneration, Fundus photography, Clinical validation, Image processing, Retinal diseases

## Abstract

Age-related macular degeneration (AMD) is a leading cause of blindness worldwide. Early detection is essential for implementing preventative measures that can slow or stop the progression of late AMD. This study evaluates the performance of an AI-based system designed to detect referable AMD, defined as more than early AMD (mteAMD), in adults over 50 who have not been previously diagnosed. Using color fundus photographs, we recruited 845 subjects from three primary care and three general ophthalmology clinics in New York City. Non-dilated images of both eyes were taken, and for validation, dilated images were reviewed by three ophthalmologists who classified the cases as no AMD, early, intermediate, or late AMD. The system’s performance was assessed on both a per-patient and per-eye basis, comparing its results to expert gradings using metrics such as AUC, sensitivity, specificity, positive predictive value, and negative predictive value. For identifying mteAMD at the subject level, the AI system achieved an AUC of 0.92, with a sensitivity of 90.27% and specificity of 83.36%, demonstrating its strong potential for early diagnosis and screening of AMD in real-world clinical settings.

**Supplementary Information:**

The online version contains supplementary material available at 10.1038/s41598-025-94866-6.

## Introduction

**Fig. 1:**
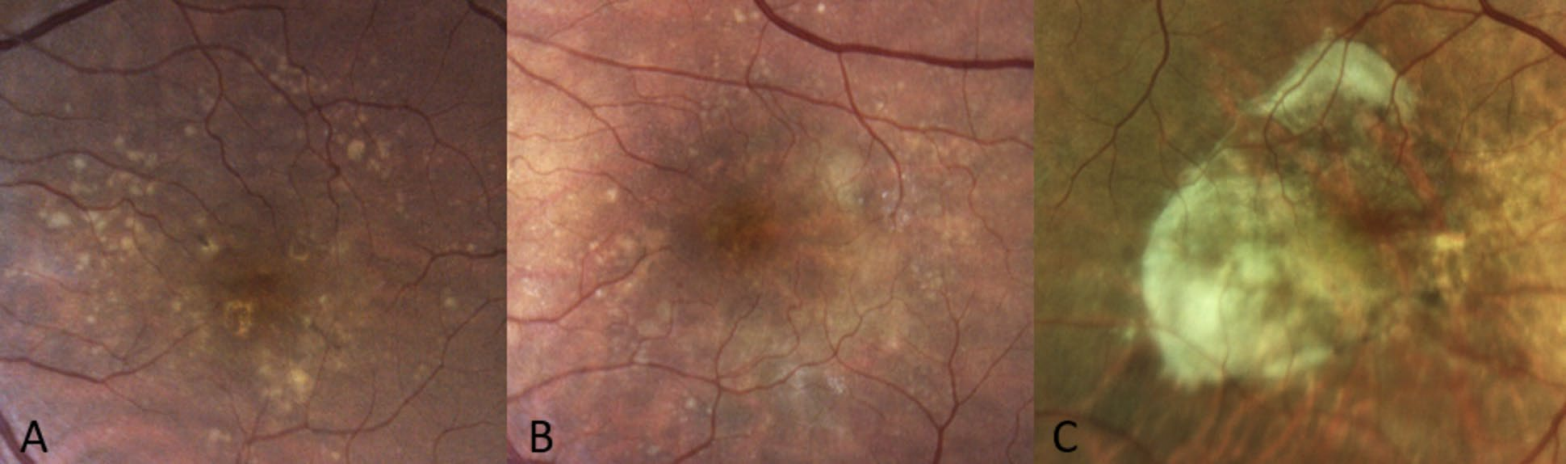
Representative retinal features from fundus imaging from our study (**A**) Soft drusen (**B**) Intermediate AMD showing hard drusen. (**C**) Geographic atrophy.

Age-related macular degeneration (AMD) is a leading cause of blindness in developed countries. It is estimated that by 2040, around 288 million people will be affected by this disease^1–4^. The number of people with AMD is expected to increase 1.5-fold over ten years due to the aging population and other causes^5–8^. Treatment for advanced disease with intraocular injections, when available, is both expensive and resource-intensive^9^. Such treatment may also be associated with cardiovascular risks^10^ or even result in Geographic Atrophy (GA)^11^. The Age-Related Eye Disease Study (AREDS) demonstrated that early identification of intermediate AMD (large drusen or many intermediate drusen) would be beneficial because of treatment that can decrease progression to late-stage AMD. It is a progressive eye disease characterized by retinal features such as drusen deposits, geographic atrophy, and/or choroidal neovascularization, leading to central vision loss (see Fig. 1). Indeed, treatment with AREDS2 vitamins, which consist of antioxidants and minerals, confers approximately a 25% reduction in progression to late AMD over 5 years^12–14^. Thus, regular screening to identify individuals with intermediate AMD was suggested to prevent progression to sight-threatening late AMD.

The National Eye Institute (NEI), USA recommends regular eye examinations by ophthalmologists for individuals above 55 years of age with other AMD risk factors^15^. However, despite these recommendations, such regular screenings are not being done^16^. This is because of the lack of automated screening tool which could enable the AMD screening in the primary care settings for the patients who are not compliant to the opthalmologists’ visit despite the ophthalmologists visit recommendation. For diabetic retinopathy, a similar problem persists. An NEI report showed that half of diabetic patients do not visit ophthalmologists regularly as advised by their primary care physicians (PCPs)^17^. A solution to this problem, and perhaps for AMD also, would be screening in the primary care setting itself, without the need for initial travel to the specialist’s office. The DR screening in the primary care settings is now well established.

Some feasibility studies have demonstrated a substantial benefit of AI-based systems for diabetic retinopathy (DR) screening in primary care^18–24^. Given the overlap between screening workflows for diabetic retinopathy (DR) and age-related macular degeneration (AMD), it is important to consider advancements in AI-based systems for DR detection. Rajesh et al. (2023) demonstrated the effectiveness of a prospectively validated AI algorithm for DR detection, achieving sensitivity and specificity rates exceeding 85%. Our team at iHealthScreen also previously published a method for combined AMD and DR screening using the same images with promising clinically acceptable results^25^. These advancements in AI for DR highlight the potential for similar applications in AMD screening, where the development of accurate, efficient, and scalable tools is critical for early diagnosis and intervention. Our study builds upon these efforts, applying ensemble deep learning techniques optimized for AMD detection in diverse clinical settings.

However, though they are needed for AMD in light of its high prevalence and social impact, such screening programs, developed with large-scale datasets and externally validated prospectively, are virtually nonexistent^26^. One of the earliest works in automated AMD screening systems used pre-deep learning statistical techniques that cannot be used in large-scale screening today due to their lack of validation and inherent weaknesses of image processing^27^. Chan et al.. showed the cost-effectiveness of screening *simultaneously*for intermediate AMD in patients already being screened for diabetic retinopathy^16^.

Many AI-based tools have been developed to improve the detection and classification of age-related macular degeneration (AMD) using various deep learning techniques and different imaging methods. For example, the Multi-modal, Multi-task, Multi-attention framework combines color fundus photography (CFP) and fundus autofluorescence (FAF) images and achieves an area under the curve (AUC) of 0.94 in detecting reticular pseudodrusen (RPD), which is an important feature of AMD^28^. Another tool is a Multi-Scale Convolutional Neural Network (CNN) that works on retinal optical coherence tomography (OCT) images, showing high performance with an AUC of 0.96 for AMD classification^29^. One more approach uses weakly-supervised learning to find lesions in fundus images, reaching more than 85% accuracy in lesion localization and giving explainable results that help doctors understand the findings^30^. Also, an automated AMD area estimation method, which uses ResNet50 and UNet models, shows a Dice coefficient of 0.83 when compared with expert-annotated images (arXiv:2107.02211). These research schemes were based on the retrospective datasets and did not focus on the prospective study in the clinical settings which we are addressing in this paper.

We developed an AI-based AMD screening system to screen subjects at any stage and refer the mteAMD to a retinal specialist. In this paper, we report the results of this prospective study demonstrating the efficacy of this system in primary care and ophthalmology settings.

## Study design and methods

### Prospective study design

#### Compliance with guidelines and ethical approval

All methods were carried out in accordance with relevant FDA guidelines and regulations governing clinical trials for medical devices. The experimental protocols were reviewed and approved by *Mount Sinai Institutional Review Board (IRB)*, ensuring adherence to ethical standards. Informed consent was obtained from all subjects, and where applicable, from their legal guardian(s), prior to participation in the study.

*Icahn School of Medicine at Mount Sinai Institutional Review Board (IRB) approved the trial protocol (IRB-18–00778) and also registered it with clinicaltrials.gov (NCT # 04863391).*

**Fig. 2:**
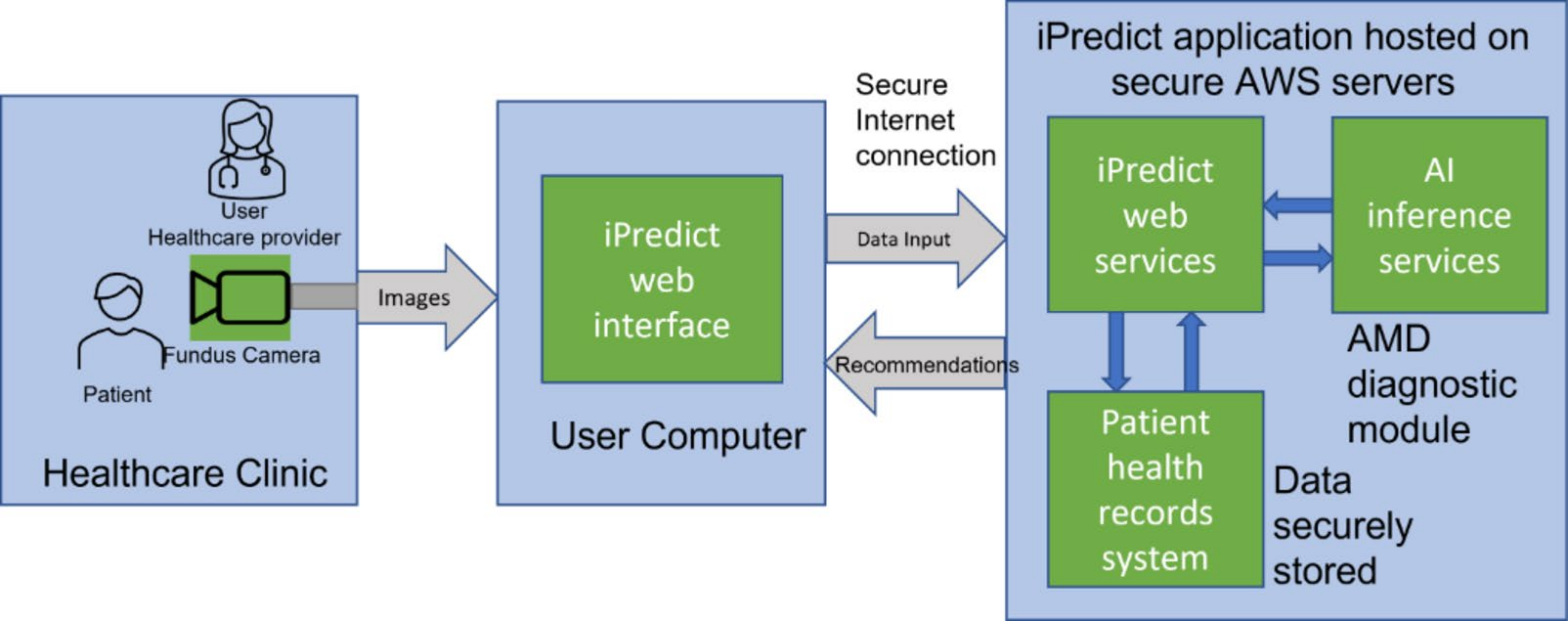
Various modules in the iPredict-AMD screening system.

The automated AI-based AMD screening tool (see Fig. 2), iPredict-AMD, was validated in a prospective, multi-center clinical study. 845 subjects were enrolled from six clinics (3 primary care and 3 ophthalmology), 411 subjects from primary care, and 434 from ophthalmology clinics before exclusion and dropouts.

Inclusion criteria: Subjects were over age 50, without any history of any form of AMD.

Exclusion criteria: Subjects who were unable to consent to the study, unable to undergo study procedures, with known diagnosis of other retinal diseases such as retinal vein occlusion or diabetic retinopathy, or were participating in other AMD trials involving investigational products were excluded.

## Study procedures

By analyzing subjects’ retinal images, the system (iPredict-AMD) automatically identified those who had more than early AMD or “mteAMD,” i.e., those at risk of progression to late AMD, in these steps:

- Retinal images of both eyes were captured without pupil dilation for AI evaluation with the fully automated DRSPlus color fundus camera (from iCare Inc.). For ground truth evaluation, the pupil-dilated images were taken by the trained imaging operator, and evaluated by 3 ophthalmologists to compare the AI-generated results.
- All fundus images captured using the DRSPlus color fundus camera maintain a consistent field of view (FOV) across all images to ensure uniformity in image quality and parameter extraction.
- One fundus image per eye was captured for each patient (for AI device input), ensuring consistency across all study subjects in data collection and analysis.
- Retinal images were uploaded to the automated AI system iPredict-AMD to identify those with more than early age-related macular degeneration (mteAMD), i.e., referable AMD.
- The system generated an automated report (within a minute). If referable AMD was identified, it advised the physician to refer the patient to an ophthalmologist; or otherwise, to advise the patient to return in one year.

## AI module

AI is being used extensively in medicine, especially in retinal diseases, with promising results^26,31–36^. The iPredict-AMD AI module (deep machine learning and decision modules) is built from five deep learning architectures, deployed in cloud servers, that are ensembled to produce a vector output^37,38^. This output is optimized in the inference models and returns the result as mteAMD detected (referable) or non-referable AMD to the webserver for report generation. Details are in the supplementary material.

Briefly, the uploaded fundus image (after undergoing a quality assessment step to ensure suitability for analysis; the image quality assessment step involves an independent deep learning model and determines if the image quality is sufficient for automatic evaluation) undergoes preprocessing (local color averaging) and resizing into the resolutions needed to fit the five trained networks which are based on Xception, Inception-V3, and Inception-Resnet-V2 architectures, the details on the DL models can be obtained from our previous paper on the system design^37^. The networks have each been trained to classify the image into one of the four classes of AMD (no, early, intermediate, and advanced) based on the AREDS 4-level AMD category scale^39^. Each network produces 4 probabilities, one for the image to fall into each class. All the probabilities from all the networks are then used in the weighted LMT model as an ensembling technique to produce the best fit to the data set (based on the least error to stop the training). The final score of the equation determines the overall referability of the fundus image.

The automated AI report includes the patient’s identifiable information, and color fundus images for both eyes and provides one of the following outputs for each eye:

- mteAMD detected or referable AMD in either eye (refer to an ophthalmologist) for intermediate and Late AMD subjects.
- mteAMD not detected or non-referable AMD in both eyes (and revisit for screening in one year), or.
- Insufficient image quality or referable to an ophthalmologist as output.

The model was developed and tested using deep learning (DL) techniques with 116,875 color fundus photos from 4,139 participants in the AREDS study^40^.

## Ground truth evaluation

For ground truth evaluation, pupil-dilated images were captured by a trained imaging operator and independently reviewed by three ophthalmologists. Using the gold standard (i.e., following AREDS protocol which scales no AMD, early AMD, intermediate AMD, and late AMD based on the AMD pathologies in the individual’s color fundus image), the three ophthalmologists grade the subject’s color fundus images. Each ophthalmologist graded all the images, and the majority decision was used as the ground truth for comparison with the AI-generated results. The patient information and individual gradings were masked.

### Clinical study results

The AI tool, iPredict-AMD, was tested using a prospective clinical study design involving 696 subjects across six clinical sites. Retinal images were captured using non-mydriatic fundus cameras, and the AI tool’s performance was validated by comparing its outputs against the ground truth established by three independent graders. Testing included both subject-level and eye-level evaluations, with metrics such as sensitivity, specificity, positive predictive value, and negative predictive value assessed. Details of the model training, including dataset characteristics and the AI workflow, are provided in the Supplementary Appendix.

**Enrollment.** Out of the 845 subjects, 48 failed to meet eligibility requirements due to other diseases and hence, were removed from the study. 5 participants were under the age of 50. 55 participants had a history of AMD which was not reported during initial selection. 41 of the remaining subjects did not want to continue to ground truth or did not follow protocol. 696 participants are included in the primary endpoints’ calculation, out of whom 339 were from primary care and 357 were from ophthalmology clinics. Table 1 describes the demographics of the study population. The key performance measures are summarized in Table 2.

**Table 1:** Demographic characteristics of the study population.

	Overall study population	Excluded from study
Total	696	149
Female	373	80
Male	323	69
Median age (years)	59	56
Asian American	32 (4.6%)	n/a (data incomplete)
Black/African American	158 (22.7%)	n/a (data incomplete)
Hispanic of any race	206 (29.6%)	n/a (data incomplete)
Native American/Alaskan native	44 (6.3%)	n/a (data incomplete)
Native Hawaiian/Pacific Islander	7 (1.0%)	n/a (data incomplete)
Non-Hispanic white	231 (33.2%)	n/a (data incomplete)
Other (s)	18 (2.5%)	n/a (data incomplete)

**Table 2:** Summary of performance of the AI system (Primary endpoints subject-wise) for the cohorts at all sites. All ranges are 95% confidence intervals (CI).

	Overall study population (*N* = 696 subjects, 1392 eyes) mteAMD grading		
Reference std grading (below)	Pos	Neg	Total
Positive	102	11	113
Negative	97	486	583
Total	199	497	696
Sensitivity	90.27% (83.25–95.04%)		
Specificity	83.36% (80.09–86.30%)		
Positive likelihood ratio	5.43 (4.48 to 6.57)		
Negative likelihood ratio	0.12 (0.07 to 0.20)		
Positive predictive value (PPV)	51.26% (46.47–56.02%)		
Negative predictive value (NPV)	97.79% (96.18–98.73%)		
Disease prevalence	16.24% (13.57–19.19%)		

**Fig. 3:**
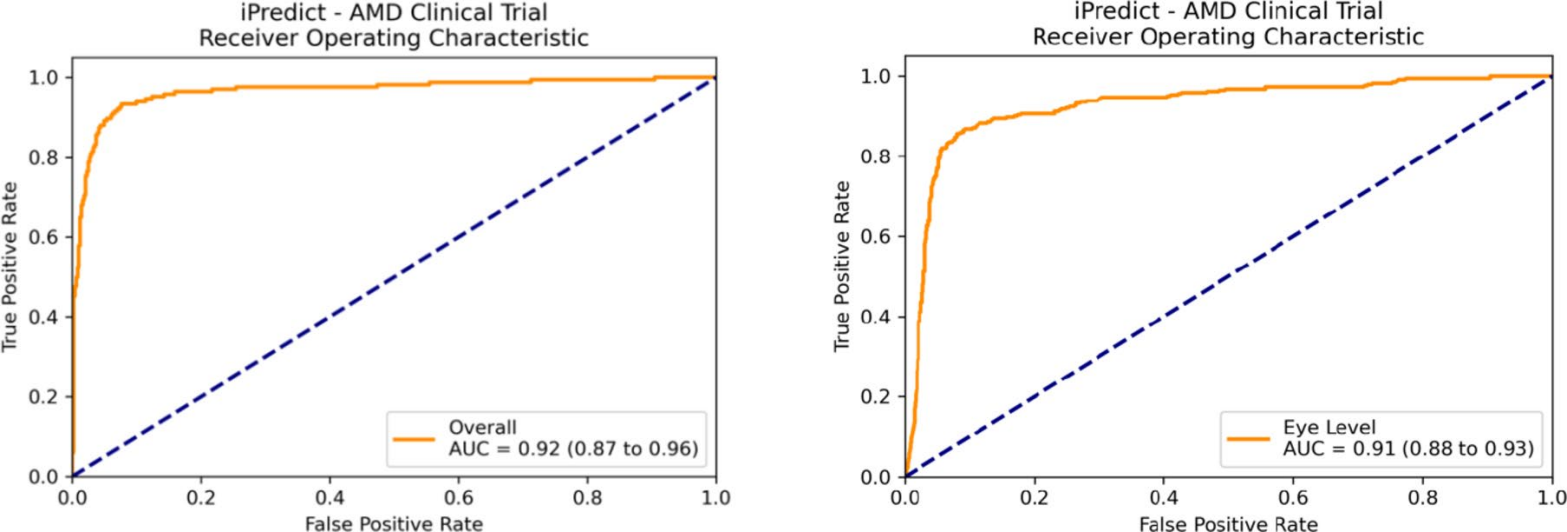
Receiving operating characteristics for the performance analysis of iPredict-AMD for the overall population - subject level (left) and eye-level (right) evaluation of the device.

Table 2 shows the Performance (Primary Endpoints) at the subject level for the Cohorts at all Sites. Table 3 shows the performance at the eye level. When considering the overall study population, the iPredict-AMD system showed a sensitivity of 90.27% and a specificity of 83.36%. Figure 3 shows the AUCs for the subject-level and eye-level performance in the overall population.

**Table 3:** Eye-level MteAMD detection for the subjects. All ranges are 95% confidence intervals (CI).

	Overall study population (1392 eyes) mteAMD grading		
Reference Std grading (below)	Pos	Neg	Total
Positive	203	26	229
Negative	196	967	1163
Total	399	993	1392
Sensitivity	88.65% (83.81–92.45%		
Specificity	83.15% (80.87–85.26%)		
Positive likelihood ratio	5.26 (4.59 to 6.03)		
Negative likelihood ratio	0.14 (0.09 to 0.20)		
Positive predictive value (PPV)	50.88% (47.48–54.26%)		
Negative predictive value (NPV)	97.38% (96.28–98.16%)		
Disease prevalence	16.45% (14.54–18.51%)		

## Discussion and conclusions

iPredict-AMD is indicated for use by health care providers (physicians and/or their assistants) to automatically detect more than early AMD (mteAMD) in adults above 50 years of age who have not been previously diagnosed with AMD. The system was tested and validated against an external retrospective dataset (NAT2 dataset) and the results were reported in an earlier paper^41^. This prospective clinical study demonstrates that iPredict-AMD has consistent accuracy and a clinically meaningful benefit in the detection of more than early AMD.

The device’s observed performance facilitated earlier detection of AMD (i.e., mteAMD or referable AMD) for 102 subjects out of 113 subjects correctly. We note that the other 11 subjects were advised to have yearly screening. This will ensure that the borderline referable AMD subjects who were missed at this time will be potentially identified at the next screening. Also, we observed that a good number of eyes with concomitant diseases were detected as referable, possibly due to the retinal features seen as not normal by the model. The model was not designed or trained to detect diseases other than AMD. However, we had 24 participants with referable DR (detected by the graders) of which 15 were identified as referable by the system. The other 9 subjects with moderate DR who were missed by our AMD screening tool may be screened for diabetic retinopathy as per the yearly DR screening protocol. We tested our DR screening tool (a similar retinal disease screening tool built in-house) on these subjects and all 24 participants were detected as referable DR correctly. In addition to DR, we had 5 participants with “epiretinal membrane”, 6 participants with High Myopia, and 8 participants with “macular pucker”, and all of them were identified as referable by our AMD screening tool. We believe this to be an added benefit of detecting some concomitant diseases early, even though the model is not designed to detect them. Furthermore, the excellent Negative Predictive Value of the study, 97.79%, confirms that of all those screened negative for referable AMD, almost all would be found not to have iAMD if referred. The corresponding low false negative rate is an excellent safety standard for the larger study population. Several AI-based systems for AMD screening have been developed and validated. For instance, a study by Liefers et al. (2021) prospectively validated an AI-based system for detecting AMD in a clinical setting, demonstrating its feasibility and diagnostic accuracy (Liefers et al., Acta Ophthalmologica, 2021). Their model achieved high sensitivity and specificity, comparable to human graders. In comparison, our system leverages ensemble deep learning architectures and incorporates a unique workflow optimized for both primary care and ophthalmology clinics, achieving robust performance metrics such as an AUC of 0.92. This positions our approach as a versatile and highly accurate tool for referable AMD detection.

Regarding cost, the low-cost non-mydriatic fundus camera is easily operable by office staff, as is photo uploading for AI interpretation. Thus, AI-based screening will be accessible to primary care physicians and other healthcare professionals, who may see patients at risk for AMD but are not under routine eye specialty care. This will allow the earlier diagnosis of AMD through yearly screening, eye specialist follow-up, and initiation of therapy, if necessary, as suggested by Chew and Schachat (2015)^16^. The modest expense of the screening program will be significantly offset by future savings in patients’ vision and healthcare costs. A key strength of our approach lies in its use of non-mydriatic imaging and rapid AI analysis, which facilitate deployment in diverse clinical settings. However, potential barriers to adoption in rural or low-resource clinics should be considered. These barriers may include limited access to high-quality imaging devices, insufficient internet bandwidth for cloud-based AI processing, and a lack of trained personnel for basic system operation. One possible reason could be the absence of an automated screening tool to facilitate AMD screening in primary care for patients who do not follow ophthalmologists’ visit recommendations. Addressing these challenges could involve developing portable imaging devices, providing offline AI processing capabilities, and implementing targeted training programs for clinical staff.

Future integration of AI screening tools with electronic health record (EHR) systems represents another important avenue for enhancing real-world deployment. Seamless integration with EHR systems could enable automated reporting, streamlined referrals, and improved tracking of patient outcomes, ultimately increasing the efficiency and effectiveness of AMD screening programs. Further studies focusing on these integration strategies will be crucial for scaling AI-driven AMD detection to broader populations.

The AREDS study, the largest AMD study in the world, showed that specific vitamins and minerals, along with lifestyle changes, can reduce the risk of progression of intermediate AMD to late AMD and improve visual acuity in approximately 25% of patients^13^. Identification of individuals with mte or intermediate AMD is, therefore, crucial to providing prognostic counseling or initiating treatment with these minimal-risk interventions. With an AMD screening tool in primary care clinics, asymptomatic early and intermediate stages of AMD in a broader population might be detected earlier, with potentially better outcomes.

Some limitations include subject dropouts and the study being conducted in a single geographical location, which may impact generalizability. The dropout reasons (e.g., subjects did not want to continue to ground truth imaging, fell below the age limit, failed to meet prespecified eligibility criteria, etc.) and the numbers are shown as a flow chart in the Appendix. However, these should not be a concern for the following reasons. The dropouts were random for age, gender, and race and did not affect the final statistical evaluation. Also, the study focused on the prospective validation of a medical device that follows the FDA’s safety and efficacy guidelines. A similar screening tool, AEye-ds (FDA device number K221183), has been FDA-approved and was tested prospectively on fewer than 500 subjects^42^. The study was conducted in New York City with diverse demographics and healthcare systems, the recruited subjects represent a diverse population group.

There are other benefits for patients. This screening does not need dilation before imaging because iPredict-AMD uses a non-mydriatic camera, DRSPlus. Dilation often causes patient discomfort and is generally only performed by eye-care professionals and requires a longer stay in the clinic. The increased time requirement and sensitivity to the light with dilated pupils can reduce patient willingness to comply with the screening. Additionally, dilation can cause increased intraocular pressure, a risk factor for glaucoma^43^. The proposed AMD analysis tool used in conjunction with the DRSPlus camera with high-speed, fully automated, and easy-to-use features can be safely and rapidly deployed in a broad range of clinical settings, thus being an effective tool for AMD screening and blindness prevention.

## Electronic supplementary material

Below is the link to the electronic supplementary material.

Supplementary Material 1

## Data Availability

The dataset is held by iHealthscreen Inc. Upon request, de-identified data may be made available by contacting the corresponding author at bhuiyan@ihealthscreen.org.

## References

[1] Wong, W. et al. Global prevalence of age-related macular degeneration and disease burden projection for 2020 and 2040: a systematic review and meta-analysis. *Lancet Glob Health*. **2** (2), e106–e116. 10.1016/S2214-109X(13)70145-1 (2014). 25104651 10.1016/S2214-109X(13)70145-1

[2] Thomas, C. J., Mirza, R. G. & Gill, M. K. Age-related macular degeneration. *Med. Clin.* **105** (3), 473–491 (2021). 10.1016/j.mcna.2021.01.003 33926642

[3] Stahl, A. The diagnosis and treatment of age-related macular degeneration. *Deutsches Ärzteblatt Int.* **117** (29–30), 513 (2020). 10.3238/arztebl.2020.0513 PMC7588619 33087239

[4] Apte, R. S. Age-related macular degeneration. *N. Engl. J. Med.* **385** (6), 539–547 (2021). 34347954 10.1056/NEJMcp2102061 PMC9369215

[5] Wong, T. Y., Liew, G. & Mitchell, P. Clinical update: new treatments for age-related macular degeneration. *Lancet* **370**, 194–206 (2007). 10.1016/S0140-6736(07)61104-0 17658379

[6] WT, Y. Age related macular degenration: time for a randomized controlled trial. *Am. J. Ophthalmol.* **144** (1), 117–119 (2007). 17601430 10.1016/j.ajo.2007.04.018

[7] Lim, L. S., Mitchell, P., Seddon, J. M., Holz, F. G. & Wong, T. Y. Age-related macular degeneration. *Lancet* **379** (9827), 1728–1738 (2012). 22559899 10.1016/S0140-6736(12)60282-7

[8] Ahlers, C. et al. Imaging of the retinal pigment epithelium in age-related macular degeneration using polarization sensitive optical coherence tomography. *Invest. Ophthalmol. Visual Sci.* **51** (4), 2149–2157 (2010). 19797228 10.1167/iovs.09-3817 PMC3016608

[9] Fleckenstein, M. et al. Age-related macular degeneration. *Nat. Reviews Disease Primers*. **7** (1), 31 (2021). 33958600 10.1038/s41572-021-00265-2 PMC12878645

[10] Wong, T. Y. Age-related macular degeneration and cardiovascular disease in the era of Anti–Vascular endothelial growth factor therapies. *Am. J. Ophthalmol.* **148** (3), 327–329 (2009). 19703607 10.1016/j.ajo.2009.05.012

[11] Karme, M., Anti, V. E. G. F., Treatment & Dry, A. M. D. Finding the Balance. EyeNet Magazine (http://www.oorg/eyenet/article/anti-vegf-treatment-dry-amd-finding-balance)

[12] Age-Related Eye Disease Study Research Group. The relationship of dietary carotenoid and vitamin A, E, and C intake with Age-Related macular degeneration in a Case-Control study: AREDS report 22. *Arch. Ophthalmol.* **125** (9), 1225–1232 (2007). 17846363 10.1001/archopht.125.9.1225

[13] Age-Related-Eye-Disease-Study-Research-Group. A randomized, placebo-controlled, clinical trial of high-dose supplementation with vitamins C and E, beta carotene, and zinc for age-related macular degeneration and vision loss: AREDS report 8. *Arch. Ophthalmol.* **119** (10), P1417 (2001). 10.1001/archopht.119.10.1417 PMC1462955 11594942

[14] Age-Related Eye Disease Study Research Group. The relationship of dietary carotenoid and vitamin A, E, and C intake with Age-Related macular degeneration in a Case-Control study. *Arch. Ophthalmol.* **125** (9), 1225–1232 (2007). 17846363 10.1001/archopht.125.9.1225

[15] (NEI) NEI. Am I at Risk for AMD? National Institutes of Health (NIH), USA. Accessed Sep 1. (2023). https://www.nei.nih.gov/learn-about-eye-health/eye-conditions-and-diseases/age-related-macular-degeneration

[16] Chew, E. Y. & Schachat, A. P. Should we add screening of age-related macular degeneration to current screening programs for diabetic retinopathy? *Ophthalmology* **122** (11), 2155–2156 (2015). 26498078 10.1016/j.ophtha.2015.08.007 PMC11571100

[17] Diabetic-Eye-Disease. Facts About Diabetic Eye Disease. National Eye Institute. Last Accessed on Aug 30, (2022). https://nei.nih.gov/health/diabetic/retinopathy

[18] Ting, D. S. W. et al. Development and validation of a deep learning system for diabetic retinopathy and related eye diseases using retinal images from multiethnic populations with diabetes. *Jama* **318** (22), 2211–2223 (2017). 29234807 10.1001/jama.2017.18152 PMC5820739

[19] Abràmoff, M. D., Lavin, P. T., Birch, M., Shah, N. & Folk, J. C. Pivotal trial of an autonomous AI-based diagnostic system for detection of diabetic retinopathy in primary care offices. *Npj Digit. Med.* **2018/08**/28 2018;1(1):39. 10.1038/s41746-018-0040-6 10.1038/s41746-018-0040-6 PMC6550188 31304320

[20] Bhuiyan, A. et al. Development and validation of an automated diabetic retinopathy screening tool for primary care setting. *Diabetes Care* **43**(10), e147–e148 (2020). 32855159 10.2337/dc19-2133 PMC7510034

[21] Group, D. E. R. Frequency of evidence-based screening for retinopathy in type 1 diabetes. *N. Engl. J. Med.* **376** (16), 1507–1516 (2017). 28423305 10.1056/NEJMoa1612836 PMC5557280

[22] Gulshan, V. et al. Development and validation of a deep learning algorithm for detection of diabetic retinopathy in retinal fundus photographs. *Jama* **316** (22), 2402–2410 (2016). 27898976 10.1001/jama.2016.17216

[23] Krause, J. et al. Grader variability and the importance of reference standards for evaluating machine learning models for diabetic retinopathy. *Ophthalmology* **125** (8), 1264–1272 (2018). 29548646 10.1016/j.ophtha.2018.01.034

[24] Ta, A. W. A. et al. Two Singapore public healthcare AI applications for National screening programs and other examples. *Health Care Sci.* **1** (2), 41–57 (2022). 38938890 10.1002/hcs2.10 PMC11080681

[25] Bhuiyan, A., Govindaiah, A., Alauddin, S., Otero-Marquez, O. & Smith, R. T. Combined automated screening for age-related macular degeneration and diabetic retinopathy in primary care settings. *Annals Eye Sci.* **6**, 12 (2021). 10.21037/aes-20-114 PMC8525840 34671718

[26] Pead, E. et al. Automated detection of age-related macular degeneration in color fundus photography: a systematic review. *Surv. Ophthalmol.* **64** (4), 498–511 (2019). 30772363 10.1016/j.survophthal.2019.02.003 PMC6598673

[27] Kankanahalli, S., Burlina, P. M., Wolfson, Y., Freund, D. E. & Bressler, N. M. Automated classification of severity of age-related macular degeneration from fundus photographs. *Investig. Ophthalmol. Vis. Sci.* **54** (3), 1789–1796 (2013). 23361512 10.1167/iovs.12-10928

[28] Chen, Q. et al. Multimodal, multitask, multiattention (M3) deep learning detection of reticular Pseudodrusen: toward automated and accessible classification of age-related macular degeneration. *J. Am. Med. Inform. Assoc.* **28** (6), 1135–1148 (2021). 33792724 10.1093/jamia/ocaa302 PMC8200273

[29] Sotoudeh-Paima, S., Jodeiri, A., Hajizadeh, F. & Soltanian-Zadeh, H. Multi-scale convolutional neural network for automated AMD classification using retinal OCT images. *Comput. Biol. Med.* **144**, 105368 (2022). 35259614 10.1016/j.compbiomed.2022.105368

[30] Morano, J. et al. Weakly-supervised detection of AMD-related lesions in color fundus images using explainable deep learning. *Comput. Methods Programs Biomed.* **229**, 107296 (2023). 36481530 10.1016/j.cmpb.2022.107296

[31] Dow, E. R. et al. From data to deployment: the collaborative community on ophthalmic imaging roadmap for artificial intelligence in age-related macular degeneration. *Ophthalmology* **129** (5), e43–e59 (2022). 35016892 10.1016/j.ophtha.2022.01.002 PMC9859710

[32] Burlina, P. M. et al. Use of deep learning for detailed severity characterization and Estimation of 5-Year risk among patients with Age-Related macular degeneration. *JAMA Ophthalmol.* **136** (12), 1359–1366 (2018). 30242349 10.1001/jamaophthalmol.2018.4118 PMC6583826

[33] Fleming, A. D. et al. Deep learning detection of diabetic retinopathy in Scotland’s diabetic eye screening programme. *Br. J. Ophthalmol.* **108**(7), 984–988 (2024). 37704266 10.1136/bjo-2023-323395

[34] Ruamviboonsuk, P. et al. Real-time diabetic retinopathy screening by deep learning in a multisite National screening programme: a prospective interventional cohort study. *Lancet Digit. Health*. **4** (4), e235–e244 (2022). 35272972 10.1016/S2589-7500(22)00017-6

[35] Pedersen, E. R. et al. Redesigning clinical pathways for immediate diabetic retinopathy screening results. *NEJM Catal. Innov. Care Deliv.* **2**(8). 10.1056/CAT.21.0096 (2021).

[36] Haug, C. J. & Drazen, J. M. Artificial intelligence and machine learning in clinical medicine, 2023. *N. Engl. J. Med.* **388** (13), 1201–1208 (2023). 36988595 10.1056/NEJMra2302038

[37] Govindaiah, A., Smith, T. & Bhuiyan, A. A New and Improved Method for Automated Screening of Age-Related Macular Degeneration Using Ensemble Deep Neural Networks. *In the proceedings of IEEE EMBC 2018*. :702–705. (2018). 10.1109/EMBC.2018.8512379 30440493

[38] Bhuiyan, A. et al. Artificial intelligence to stratify severity of Age-Related macular degeneration (AMD) and predict risk of progression to late AMD. *Translational Vis. Sci. Technol.* **9** (2), 25–25 (2020). 10.1167/tvst.9.2.25 PMC7396183 32818086

[39] Age-Related-Eye-Disease-Study-Research-Group. A simplified severity scale for Age-Related macular degeneration, AREDS report 18. *Arch. Ophthalmol.* **123** (11), 1570–1574. 10.1001/archopht.123.11.1570 (2005). 16286620 10.1001/archopht.123.11.1570 PMC1473206

[40] Age-Related-Eye-Disease-Study-Research-Group. The Age-Related eye disease study (AREDS): design implications. AREDS report 1. *Control Clin. Trials*. **20** (6), 573–600 (1999). 10588299 10.1016/s0197-2456(99)00031-8 PMC1473211

[41] Govindaiah, A., Baten, A., Smith, R. T., Balasubramanian, S. & Bhuiyan, A. Optimized prediction models from fundus imaging and genetics for late age-related macular degeneration. *J. Personalized Med.* **11** (11), 1127 (2021). 10.3390/jpm11111127 PMC8617775 34834479

[42] Efficacy and Safety of AEYE-DS Software Device for Automated Detection of Diabetic Retinopathy from Digital Fundus Images. (last accessed on May 01, 2024). https://www.clinicaltrials.gov/study/NCT04612868

[43] Kim, J. M. et al. Changes in intraocular pressure after Pharmacologic pupil dilation. *BMC Ophthalmol.* **12**, 1–5 (2012). 23017184 10.1186/1471-2415-12-53 PMC3499138

