# Supplementary material for: A prospective study for validating an automated AI-based system for detecting age-related macular degeneration in clinical settings
Source: Sci Rep. 2026 Jul 29;16:23541. doi: 10.1038/s41598-025-94866-6 (PMC13421544; doi:10.1038/s41598-025-94866-6)
Supplement: Supplementary file 1 — Supplementary Material 1 [file 41598_2025_94866_MOESM1_ESM.docx]

**SUPPLEMENTARY APPENDIX**

**AI-based system description**

The parts of the **system** are summarized here and further described in the following sections.

**iPredict**-AMD Client: A cloud-based software application’s web/user interface (UI) running on a computer with internet services, usually connected to the fundus camera, at the customer site. Using this UI, the patient’s color retinal image and patient data are transferred to the AMD diagnostic module or the AI server for AMD diagnosis, and the result is a report with the recommendation of referable or non-referable AMD. If an exam cannot be analyzed due to image quality, quality feedback is provided to help the operator acquire a high-quality image and successfully obtain a result after resubmission. If the image quality is poor and automatic evaluation is not feasible, the device will output that a referral to an ophthalmologist is recommended with the citing: ‘Referable to Ophthalmologist because of insufficient quality image’.

**iPredict**-server: **iPredict** includes a general exam analysis service delivery software package with its own set of product and software requirements. **iPredict**-server contains a web server front-end that securely handles incoming requests, a database that stores customer information, and a logging system that records information about each transaction through **iPredict**-service. **iPredict** webserver is also primarily responsible for device cybersecurity. The report generation module in the web server provides the .pdf report with the decision of the patient’s status of the disease and returns the report to the user interface with the recommendation of the referable or non-referable (to ophthalmologist) disease status of the patient.

**iPredict**-AMD AI module: The AI module (deep machine learning and decision modules) processes the images in the server and returns the results as mteAMD detected or non-referable or non-referable AMD to the webserver to produce the report generation module. Details in the Deep learning or AI algorithm section.

The model was developed and tested using deep learning techniques with 116,875 color fundus photos from 4,139 participants in the AREDS study.

Non-referable AMD refers to either no AMD (a few small or no drusen) or early AMD (many small drusen or a few medium-sized drusen in one or both eyes). Referable AMD refers to either Intermediate AMD (many medium-sized drusen or one or more large drusen in one or both eyes or Advanced AMD - either a breakdown of light-sensitive cells and supporting tissue in the central retinal area (advanced dry form) or geographic atrophy (GA) or abnormal and fragile blood vessels under the retina (wet form). There were 4305 images considered as mteAMD and 5170 considered as no mteAMD in the test dataset.

The target environment is a clinical setting with standard room settings lighting, and other conditions. The users are intended to be healthcare providers, physicians, nurses, or healthcare assistants/workers with self-training – a maximum of fifteen minutes to half an hour.

**Sample Size Calculation for the Prospective Clinical Study**

The total estimated sample size is 845 subjects for evaluation - including 189 for mteAMD subjects (for sensitivity calculations) and 656 for combined early AMD + no AMD subjects (for specificity) after losses.

The primary effectiveness endpoints for this study are sensitivity and specificity. One-sided 97.5% confidence intervals for the lower bounds will be calculated for these binary measures. Descriptive statistics will be presented for the percent positive separately for each of mteAMD and no/early AMD.

# Exact binomial test: One-sided 2.5% alpha and 85-90% power

|  | Sensitivity | Specificity |
| --- | --- | --- |
| Null hypothesis proportion | 0.750 | 0.775 |
| Alternative proportion | 0.850 | 0.825 |
| Power (%) | 85 | 90 |
| N | 189 | 656 |

Corresponding statistical significance (one-sided p=0.025) will be achieved as displayed below.

# Exact binomial test: Reaching one-sided p=0.025

|  | **Sensitivity** | **Specificity** |
| --- | --- | --- |
| **Null hypothesis proportion** | 0.750 | 0.775 |
| **Success proportion** | 0.812 | 0.807 |

**Intent to screen population breakdown**

Figure S1 shows the number of participants selected for the final study evaluation and the number of participants who were not selected for the final study.


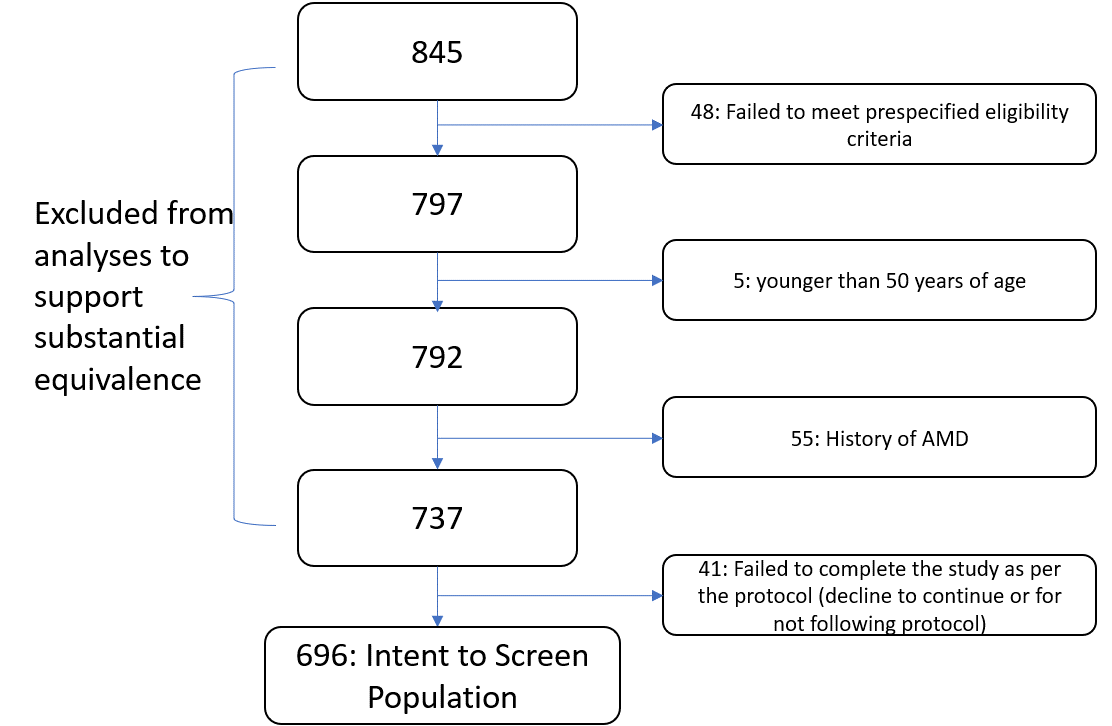


Figure S1: Intent to screen population showing the number of participants selected for the final study evaluation.

**Detailed results breakdown are shown in supplementary tables S1, S2 and S3 showing sub-analysis between ophthalmology and primary care clinics.**

**Table S1: Eye-level mteAMD detection for the subjects. All ranges are 95% confidence intervals (CI).**

|  | iPredict mteAMD output | | | | | | | | |
| --- | --- | --- | --- | --- | --- | --- | --- | --- | --- |
|  | Primary care (678 eyes) mteAMD grading | | | Ophthalmology 714 eyes) mteAMD grading | | | Overall Study Population (1392 eyes) mteAMD grading | | |
| Ground truth\ mteAMD | Pos | Neg | Total | Pos | Neg | Total | Pos | Neg | Total |
| Positive | 98 | 14 | 112 | 105 | 12 | 117 | 203 | 26 | 229 |
| Negative | 94 | 472 | 566 | 102 | 495 | 597 | 196 | 967 | 1163 |
| Total | 192 | 486 | 678 | 207 | 507 | 714 | 399 | 993 | 1392 |
| Sensitivity | 87.50% (79.92% to 92.99%) | | | 89.74% (82.77% to 94.59%) | | | 88.65%(83.81% to 92.45% | | |
| Specificity | 83.39% (80.07% to 86.37%) | | | 82.91% (79.65% to 85.85%) | | | 83.15% (80.87% to 85.26%) | | |
| Positive Likelihood Ratio | 5.27 (4.32 to 6.42) | | | 5.25 (4.36 to 6.33) | | | 5.26 (4.59 to 6.03) | | |
| Negative Likelihood Ratio | 0.15 (0.09 to 0.25) | | | 0.12 (0.07 to 0.21) | | | 0.14 (0.09 to 0.20) | | |
| Positive Predictive Value (PPV) | 51.04% (46.11% to 55.95%) | | | 50.72% (46.06% to 55.38%) | | | 50.88% (47.48% to 54.26%) | | |
| Negative Predictive Value (NPV) | 97.12% (95.38% to 98.22%) | | | 97.63% (96.02% to 98.60%) | | | 97.38% (96.28% to 98.16%) | | |
| Disease Prevalence | 16.52% (13.80% to 19.53%) | | | 16.39% (13.74% to 19.31%) | | | 16.45% (14.54% to 18.51%) | | |

**Table S2. Demographic Characteristics of the Study Population**

|  | Ophthalmology. Clinics | Primary Care Clinic | Overall Study Population |
| --- | --- | --- | --- |
| Total | 357 | 339 | 696 |
| Female | 192 | 181 | 373 |
| Male | 165 | 158 | 323 |
| Median Age (years) | 61 | 58 | 59 |
| Asian American | 19 (5.3%) | 13 (3.8%) | 32 (4.6%) |
| Black /African American | 69 (19.3%) | 89 (24.9%) | 158 (22.7%) |
| Hispanic of any race | 95 (26.6%) | 111 (31.1%) | 206 (29.6%) |
| Native American/ Alaskan native | 26 (7.3%) | 18 (5.0%) | 44 (6.3%) |
| Native Hawaiian / Pacific Islander | 7 (1.9%) | 0 (0%) | 7(1.0%) |
| Non-Hispanic White | 133 (37.3%) | 98 (27.5%) | 231(33.2%) |
| Other (s) | 8 (2.24%) | 10 (2.9%) | 18(2.5%) |

**Table S3: Summary of Performance of the AI system (Primary Endpoints subject-wise) for the Cohorts at Primary Care and Ophthalmology Sites. All ranges are 95% confidence intervals (CI).**

|  | mteAMD output | | | | | | | | |
| --- | --- | --- | --- | --- | --- | --- | --- | --- | --- |
|  | Primary care (N = 339 subjects, 678 eyes) mteAMD grading | | | Ophthalmology (N = 357 subjects, 714 eyes) mteAMD grading | | | Overall Study Population (N = 696 subjects, 1392 eyes) mteAMD grading | | |
| Ground truth\ mteAMD | Pos | Neg | Total | Pos | Neg | Total | Pos | Neg | Total |
| Positive | 49 | 6 | 55 | 53 | 5 | 58 | 102 | 11 | 113 |
| Negative | 47 | 237 | 284 | 50 | 249 | 299 | 97 | 486 | 583 |
| Total | 96 | 243 | 339 | 103 | 254 | 357 | 199 | 497 | 696 |
| Sensitivity | 89.09% (77.75% to 95.89%) | | | 91.38% (81.02% to 97.14%) | | | 90.27% (83.25% to 95.04%) | | |
| Specificity | 83.45% (78.61% to 87.58%) | | | 83.28% (78.56% to 87.33%) | | | 83.36% (80.09% to 86.30%) | | |
| Positive Likelihood Ratio | 5.38 (4.08 to 7.10) | | | 5.46 (4.19 to 7.12) | | | 5.43 (4.48 to 6.57) | | |
| Negative Likelihood Ratio | 0.13 (0.06 to 0.28) | | | 0.10 (0.04 to 0.24) | | | 0.12 (0.07 to 0.20) | | |
| Positive Predictive Value (PPV) | 51.04% (44.14% to 57.90%) | | | 51.46% (44.85% to 58.01%) | | | 51.26% (46.47% to 56.02%) | | |
| Negative Predictive Value (NPV) | 97.53% (94.88% to 98.83%) | | | 98.03% (95.56% to 99.14%) | | | 97.79% (96.18% to 98.73%) | | |
| Disease Prevalence | 16.22% (12.46% to 20.59%) | | | 16.25% (12.57% to 20.49%) | | | 16.24% (13.57% to 19.19%) | | |
